# Curdlan sulphate modulates protein synthesis and enhances NF-κB and C/EBP binding activity in HepG2 cells

**DOI:** 10.1080/09629359791947

**Published:** 1997-02

**Authors:** A. Guzdek, H. Rokita

**Affiliations:** 1 Institute of Molecular Biology Jagiellonian University Al. Mickiewicza 3 Krakow 31-120 Poland

## Abstract

In human hepatoma HepG2 cell line curdlan sulphate enhances basal and interleukin-6-stimulated fibrinogen and antichymotrypsin (ACT) synthesis, slightly increases basal ceruloplasmin production and exerts only minor effects on alpha-1-proteinase inhibitor and transferrin. Curdlan sulphate may, at least in part, affect protein synthesis at a pretranslational level, as the expression of ACT mRNA was found to be increased, whereas intracellular enzyme, manganese superoxide dismutase mRNA level was decreased in the cell culture treated with curdlan sulphate. Gel mobility shift analysis revealed that curdlan sulphate increases the DNA binding activity of NF-κB and C/EBP, suggesting that these transcription factors may participate in the regulatory effects of curdlan sulphate in HepG2 cells.


Mediators of Inflammation, **6**, 58–63 (1997)

### Curdlan sulphate modulates protein synthesis and enhances NF-κB and C/EBP binding activity in HepG2 cells

A. Guzdek[CA] and H. Rokita

Institute of Molecular Biology, Jagiellonian University, 31-120 Krakow, Al. Mickiewicza 3, Poland

[CA]Corresponding Author
Tel: (+48) 12 342004
Fax: (+48) 12 336907

**In human hepatoma HepG2 cell line curdlan sulphate enhances basal and interleukin-6-stimulated fibrinogen and antichymotrypsin (ACT) synthesis, slightly increases basal ceruloplasmin production and exerts only minor effects on alpha-1-proteinase inhibitor and transferrin. Curdlan sulphate may, at least in part, affect protein synthesis at a pretranslational level, as the expression of ACT mRNA was found to be increased, whereas intracellular enzyme, manganese superoxide dismutase mRNA level was decreased in the cell culture treated with curdlan sulphate. Gel mobility shift analysis revealed that curdlan sulphate increases the DNA binding activity of NF-κB and C/EBP, suggesting that these transcription factors may participate in the regulatory effects of curdlan sulphate in HepG2 cells.**

**Key words:** C/EBP, Curdlan sulphate, MnSOD mRNA, NF-κB, Serum protein synthesis

### Introduction

Host defence potentiators, substances which improve host homeostasis, act mainly by restoration or augmentation of the host-cell responsiveness to many bioactive factors, e.g. hormones, cytokines. They stimulate the maturation and differentiation of several cells (e.g. macrophages, T-cells) important for host-defence mechanism.[1] Many polyglucans of plant, fungal and microbial origin belong to this group.[2–5] Some of them manifest antitumour, antimetastatic and anti-infectious activity, protect against cancer recurrence and carcinogenesis, and are used clinically as immunopotentiators in cancer therapy.[5–7] However, the cellular and molecular mechanisms of these immune response modifications are still not understood. A number of investigators have reported the existence of a specific β-glucan receptor on phagocytes,[8–10] NK[11] and microglial cells.[12] The binding of (1 → 3)-β-D-glucans to these receptors is thought to be the first step in mediating the effect of these polyglucans,[13–15] but the precise nature of the β-glucan receptor has not been elucidated. The best known member of the β-glucan family, lentinan, was isolated by Chihara *et al.*[1] from an edible mushroom known as a folk remedy against cancer in Japan and China. Its antitumour and antimetastatic effectiveness was confirmed in the therapy of stomach, breast and colorectal cancer.[5] The biological activities of β-glucans vary because of the differences in their physicochemical properties, such as the length of polyglucans, degree of branching, high order structure, micelle formation and substitutions.[2,14,16–18] Sulphated polysaccharides including curdlan sulphates (a semisynthetic linear sulphated polysaccharide of bacterial origin) are known as potent inhibitors of HIV replication *in vitro*.[19,20] Curdlan sulphate (CS) inhibits attachment of the virus to T-cells and in phase I clinical trial CS was found to be well tolerated.[21,22] CS is also considered as an alternative to heparin because of its anticoagulant activity with minimal side-effects (i.e. bleeding).[23] Curdlan application in the controlled drug delivery is also postulated.[24] In this paper the modulatory effects of CS on basal and interleukin-6 stimulated protein production in HepG2 cells were analysed. For comparison, along with several secreted plasma proteins the typical inducible intracellular enzyme (MnSOD) was assayed. Selected proteins were determined by immunoelectrophoresis, mRNAs by Northern blot hybridization and EMSA was used for the evaluation of nuclear factor NF-κB and C/EBP binding activity.

### Materials and Methods

### Reagents

Dulbecco's modified Eagle's medium (DMEM) and fetal calf serum (FCS) were from Gibco Life Technologies Inc. (Grand Island, NY); [α-$^{32}$P]dCTP and [γ-$^{32}$P]ATP were obtained from

© 1997 Rapid Science Publishers

Amersham International (Amersham, UK). Curdlan sulphate (molecular weight: $7.2 \times 10^4$, S content: 14%, water: 5.6%) was kindly provided by Ajinomoto Co., Inc., Tokyo, Japan, and human recombinant IL-6 by Dr Heinz Baumann (Roswell Park Cancer Institute, Buffalo, NY, USA). Antisera to human proteins were from ATAB (Stillwater, MI, USA), human antichymotrypsin cDNA probe and MnSOD cDNA probe were from ATCC (USA). Oligonucleotide probes were synthesized by Dr A. Okruszek, Molecular and Macromolecular Research Center, Polish Academy of Sciences, Łódź, Poland. All other reagents were from Sigma (St Louis, MO, USA).

### Tissue culture and plasma protein assay

HepG2 cells were grown at 37°C in 6-well tissue culture dishes (Costar) in DMEM supplemented with 10% FCS and antibiotics under a humidified atmosphere of 95% air and 5% $CO_2$. After they reached the stage of subconfluent monolayer, the medium was changed for serum-free DMEM containing 1 μM dexamethasone and 5 units of heparin/ml and tested factors i.e. curdlan sulphate (50 or 150 μg/ml), IL-6 (50 ng/ml) or the mixture of CS and IL-6 were added. After 24 h, the media were collected, dialysed, concentrated and the production of selected plasma proteins were measured by electroimmunoassay as described by Koj *et al.*[25]

### The isolation of RNA and Northern blot analysis

For RNA preparations HepG2 cells were cultured in 60-mm dishes (Costar) and when the cells were nearly confluent, curdlan sulphate (100 μg/ml), IL-6 (50 ng/ml) or the mixture of these two tested factors were added in serum-free DMEM. For SOD mRNA preparation, cells were harvested 6 h later, and for antichymotrypsin mRNA, 24 h after treatment. Total RNA was prepared using the phenol extraction method and LiCl precipitation.[26] Electrophoresis was performed in 1% agarose containing 20 mM MOPS, 4 mM sodium acetate, 1 mM EDTA and 2.2 M formaldehyde, and the separated RNA was transferred to Hybond-N membranes (Amersham, UK). Specific mRNAs were detected by hybridization of the membranes at 68°C in 10% dextran sulphate, 1 M NaCl and 1% SDS with EcoRI fragment of the pUC 19-ACT clone for human antichymotrypsin (ACT), or *Pst*I–*Xba*I fragment derived from pHcGAP plasmid for human GAPDH, or EcoRI fragment of the phMnSOD4 clone for human MnSOD labelled with $^{32}$P-dCTP by random priming.[27] To normalize hybridization signals for variations in loading and/or transfer, membranes were probed for GAPDH mRNA. Densitometry of the bands was performed using a computer imaging (MCID) system (Imaging Research Inc., Canada).

### Nuclear protein extraction

Nuclear extracts were prepared by the mini-extraction procedure.[28] Briefly, cells were scraped from plates 60 min after treatment with the tested factors, washed twice with cold PBS and centrifuged for 5 min at 400 × ***g***. Pelleted cells resuspended in buffer containing 10 mM HEPES (pH 7.8), 10 mM KCl, 2 mM $MgCl_2$, 1 mM DTT, 0.1 mM EDTA and 0.1 mM PMSF were incubated on ice for 15 min. Twenty-five μl of 40% Nonidet P-40 was added and, after mixing, centrifuged for 30 s at 14 000 rpm. Pelleted nuclei were suspended in buffer containing 50 mM HEPES, 50 mM KCl, 300 mM NaCl, 0.1 mM EDTA, 1 mM DTT and 0.1 mM PMSF, after 20 min centrifuged for 5 min at 14 000 rpm at 4°C and supernatants frozen in 10% glycerol. The protein concentration was determined by Lowry's method.

### EMSA

DNA mobility shift assay was carried out as described by Duyao *et al.*[29] A double-stranded oligonucleotide that contained two binding sites for NF-κB (5′-AAGTCCGGGTTTTCCCCAACC-3′) from the murine c-myc oncogene corresponding to bp-1101-1081 was used. The C/EBP oligonucleotide (5′-GATCTGGTATGATTTGTAATGGGGTAGGA-3′) was from human albumin promoter.[30] The double-stranded oligonucleotide probes used in gel shift analysis were either end-labelled using [γ-$^{32}$P]ATP and T4 polynucleotide kinase (NF-κB), or labelled with [α-$^{32}$P]dCTP and Klenow polymerase using the second 9-nucleotide long strand annealed as the primer (C/EBP). Five μg of nuclear proteins were used for incubation with 0.5 ng (c.$10^5$cpm) of labelled oligonucleotide. As competitors, a 100-fold excess of the oligonucleotide was added to the binding reaction.

### Statistical analysis

Data were analysed by paired Student's *t*-test. The statistical differences were evaluated in pairs: control–CS 50 μg/ml; control–150 μg/ml or IL-6–IL-6 + CS 50 μg/ml; IL-6–IL-6 + CS 150 μg/ml.

### Results

### Curdlan sulphate affects protein synthesis

The effects of curdlan sulphate, IL-6 and the mixture of CS and IL-6 on the concentrations of selected proteins in the medium of HepG2 cell culture are presented in Fig. 1. The dose of CS was chosen based on previous experiments (data not shown). A considerable increase in the concentration of fibrinogen (FBG) and antichymotrypsin (ACT), and moderate enhanced ceruloplasmin (CER) in the medium after incubation of HepG2 cells with CS for 24 h, is contrasted with small changes in transferrin (TRF) and alpha-1-proteinase inhibitor (API). As shown earlier by Koj *et al.*,[31] IL-6 strongly enhanced ACT and FBG production in HepG2 cells, moderately increased CER and API concentrations and slightly diminished TRF production. Curdlan sulphate modulated the effect of IL-6 on protein synthesis but it seems that the action of the two tested factors was not additive (Figs 1 and 2a). As is shown in Fig. 2, CS not only increased basal and IL-6-stimulated FBG synthesis but also partly reversed the inhibitory effect of IL-1 on FBG production (Fig. 2b) as this cytokine is known to inhibit FBG synthesis *in vitro*.[32]

The effect of CS and/or IL-6 on cellular mRNA level for ACT was measured. Antichymotrypsin mRNA level was determined by Northern blot analysis and relative quantity evaluated using scanner. Equal loading of lanes was established by hybridizing the same blot with an GAPDH cDNA probe. It was found that CS (100 μg/ml) increased (about two-fold) the amount of ACT mRNA (Fig. 3), suggesting that this β-glucan induces antichymotrypsin gene transcription in HepG2 cell line, though not as distinctly as IL-6 (Fig. 3).[33] CS, in the mixture with IL-6 (50 ng/ml) exert, however, only minor additive effect on ACT gene transcription.

### The influence of curdlan sulphate on MnSOD mRNA

The effect of CS or/and IL-6 on the expression of intracellular protein, manganese superoxide dismutase mRNA in HepG2 cells was also studied. Northern analysis showed that MnSOD mRNA was mainly expressed in the presence of IL-6 (Fig. 4), while curdlan sulphate slightly diminished basal and IL-6 stimulated expression of this mRNA (by 30%). Similar pictures were obtained in three experiments.

### Effect of curdlan sulphate on the binding activity of transcription factors

To examine the involvement of transcription factors in the regulatory effects of curdlan sulphate the nuclear extracts prepared from untreated, CS-, IL-6- or CS + IL-6- treated HepG2 cells were subjected to EMSA. The results

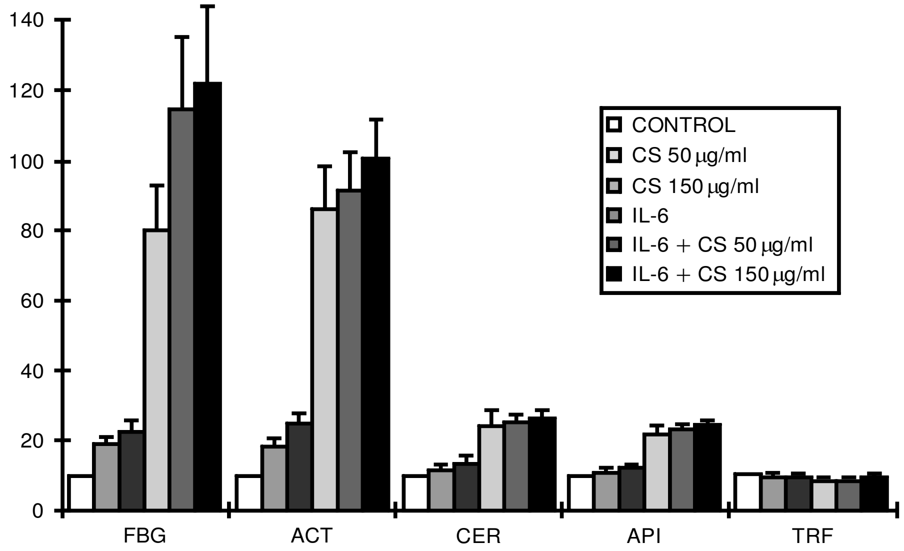

FIG. 1. Effect of curdlan sulphate (CS) on basal and interleukin-6-stimulated production of fibrinogen (FBG), antichymotrypsin (ACT), ceruloplasmin (CER), alpha-1-proteinase inhibitor (API) and transferrin (TRF) in HepG2 cells cultured for 24 h with the drug. The results are expressed in relative terms, protein content in control culture was assumed as 100. Mean of three to four experiments, ±SD. In condition used, control culture cells produced: 14.1 μg FBG, 2.1 μg ACT, 0.6 μg CER and 9.3 μg API. The increase in basal and IL-6 stimulated FBG and ACT production in comparison, respectively, with control or IL-6 treated culture is statistically significant ($P < 0.01$). Basal CER synthesis in the presence of CS (150 μg/ml) is also significantly different ($P < 0.05$) from the value for control.

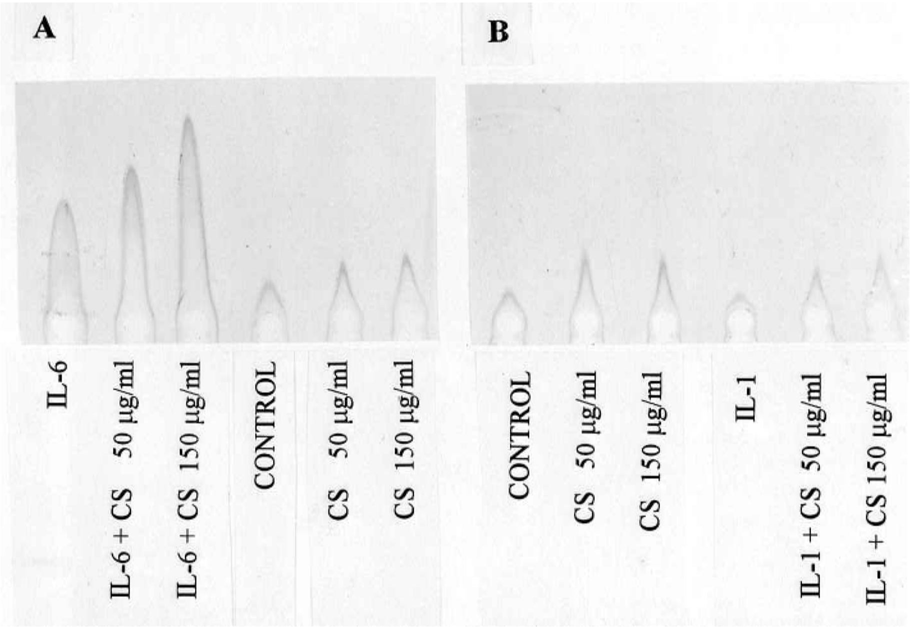

FIG. 2. Rocket immunoelectrophoresis of fibrinogen (FBG) produced by HepG2 cells cultured for 24 h with curdlan sulphate (CS) and/or IL-6 (a); CS and/or IL-1 (b).

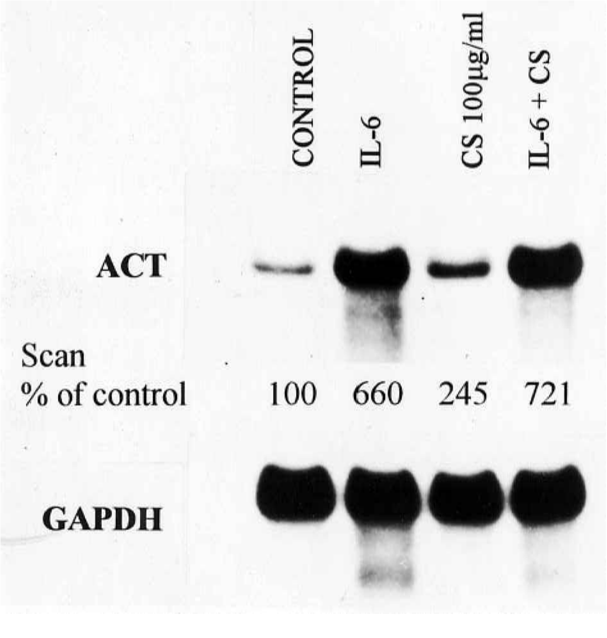

FIG. 3. The influence of curdlan sulphate (CS) (100 μg/ml) and/or IL-6 (50 ng/ml) on antichymotrypsin (ACT) gene expression (ACT mRNA) in HepG2 cells after 24 h incubation. As control, the same blot was hybridized with a GAPDH cDNA probe. The autoradiogram was densitometrically scanned, and the amount of ACT mRNA was expressed in arbitrary units, after normalizing against GAPDH mRNA control.

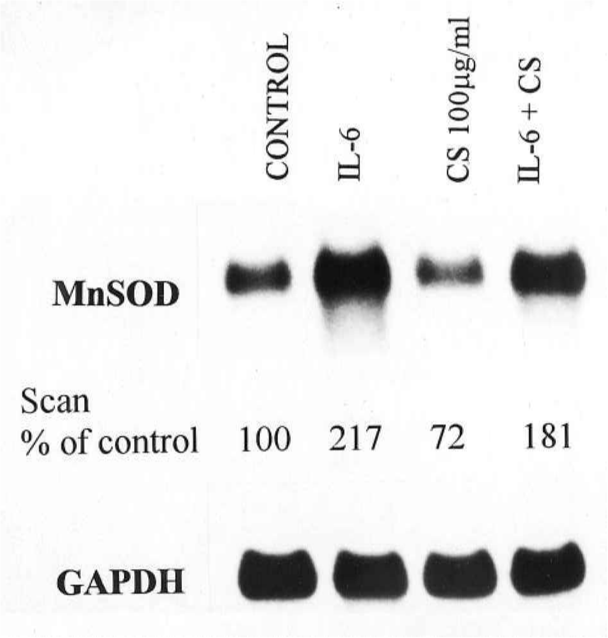

FIG. 4. The influence of curdlan sulphate (100 μg/ml), IL-6 (50 ng/ml) and the mixture of these factors on MnSOD mRNA in HepG2 cells cultured for 8 h. For comparison, mRNA for GAPDH is given. The results of one of three different experiments is shown. Scanning data are presented in arbitrary units.

depicted in Fig. 5 show that CS-induced NF-κB binding to DNA in HepG2 cells more efficiently than IL-6 and the combined effect of these drugs were not additive. We found that CS also slightly enhanced binding of transcription factors belonging to the C/EBP family in HepG2 cells.

### Discussion

Several years ago Maeda *et al.*[41] found that lentinan administration to the mouse resulted in an increase of three types of serum proteins. They observed a close relationship between the antitumour activity of this β-glucan and enhancement of protein components. Subsequent

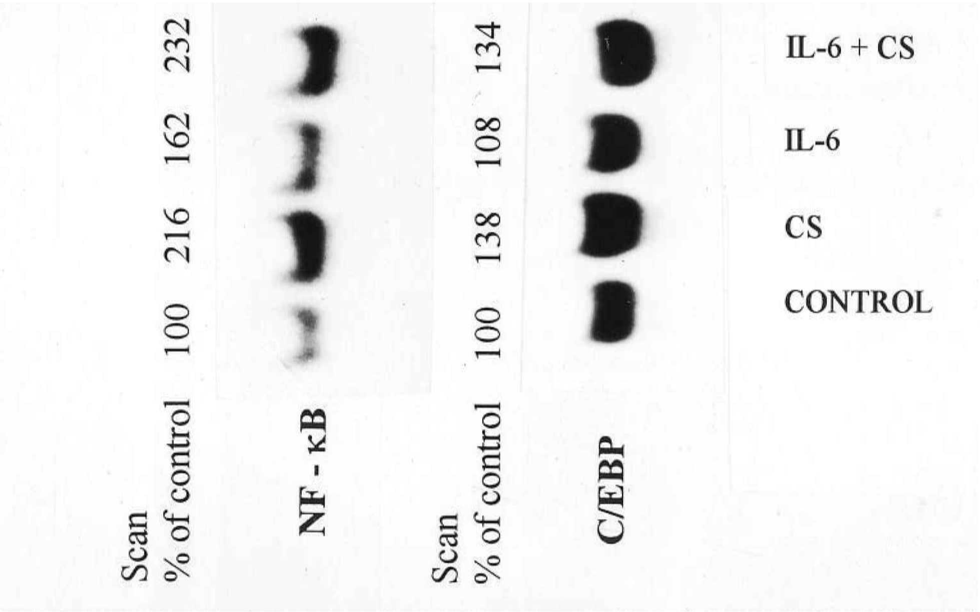

FIG. 5. Electrophoretic mobility shift assay of nuclear extracts isolated from control, curdlan sulphate- (100 μg/ml), IL-6- (50 ng/ml), or CS+IL-6-treated HepG2 cells after 60 min incubation. Double-stranded oligonucleotides, NF-κB and C/EBP were described in Materials and Methods. Densitometry scans are expressed in arbitrary units. Typical example of three experiments.

studies revealed that lentinan stimulated the production of acute phase proteins (APPs) *in vivo*.[42] The APP response to lentinan was of delayed type, genetically controlled and lentinan acted at certain optimal concentration. In our *in vitro* studies CS, another β-glucan, also induced APPs in hepatoma cells and diminished intracellular enzyme MnSOD mRNA level.

The experiments described here demonstrate that CS influence protein synthesis and transcription factors binding activity in human hepatoma HepG2 cells. Among the proteins tested, especially influenced by curdlan sulphate is fibrinogen, and, to a lesser extent, antichymotrypsin. It appears, that CS may modulate protein synthesis, at least in part, at the pretranslational level, and some of its effects are probably mediated by transcription factors NF-κB and C/EBP. The transcriptional regulation of serum protein genes is extremely complex and different cellular or environmental stimuli can distinctly alter their expression.[31,34] The most important role in the induction of serum protein genes is exerted by interleukin 6.[35,36] The induction is mediated by interleukin-6 response elements (IL-6 RE I and II). IL-6 RE I can interact with the proteins of C/EBP family and it is postulated, that this transcription factor as well as NF-κB play a regulatory role in the enhanced hepatic and cytokine-induced expression of serum protein genes.[37–39] Moreover, the synergy between NF-κB and C/EBP transcription factor families in the activation of some protein genes has been documented.[40]

Host defence potentiators affect the host metabolism, but are not cytotoxic to the tumour. It is possible, however, that CS and other bioactive polyglucans can influence protein synthesis in host and in hepatomas as well. It would be of interest to compare CS effects on control liver cells and hepatoma cells. Following this notion, the inhibition of MnSOD mRNA expression by CS may contribute to its antitumour effect, as MnSOD is one of the major cellular defence enzymes which protects superoxide radicals from toxic effects.[43] The 3′-flanking region of the MnSOD gene contains one NF-κB consensus sequence, which suggests that this potential regulatory element may play a role in the up-regulation of human MnSOD gene expression.[44] Since CS increases NF-κB binding activity and simultaneously down-regulates MnSOD gene, other mechanism(s) are possibly involved in the inhibition of MnSOD mRNA by CS. Borello *et al.*[45] recently showed that metals (iron, copper) are involved in the regulation of the MnSOD gene in rat liver. Thus the influence of CS on serum proteins, including the proteins which participate in metal distribution might be of importance.

In summary, we have shown that sulphated polyglucan, curdlan sulphate, can change protein synthesis, at least in part, at the pretranslational level, probably through the activation of NF-κB and C/EBP in the human hepatoma HepG2 cell line. It should be noted that observed alterations caused by CS are not dramatic, but it seems that biological response modifiers should act in a moderate way.

### References

1. Chihara G. Immunopharmacology of lentinan a polysaccharide isolated from *Lentinus edodes*: its application as a host defence potentiator. *Int J Oriental Med* 1992; **17**: 57–77.
2. Nemoto J, Ohno N, Saito K, Adachi Y, Yadomae T. Expression of interleukin 1 family mRNAs by a highly branched (1 → 3)-β-D-glucan, OL-2. *Biol Pharm Bull* 1993; **16**: 1046–1050.
3. Adachi Y, Okazaki M, Ohno N, Yadomae T. Enhancement of cytokine production by macrophages stimulated with (1 → 3)-β-D-glucan, grifolan (GRN), isolated from *Grifolia frondosa*. *Biol Pharm Bull* 1994; **17**: 1554–1560.
4. Yoshida I, Kiho T, Usui S, Sakushima M, Ukai S. Polysaccharides in fungi. 37. Immunomodulating activities of carboxymethylated derivatives of linear (1 → 3)-alpha-D-glucans extracted from the fruiting bodies of *Agrocybe cylindracea* and *Amanita muscaria*. *Biol Pharm Bull* 1996; **19**: 114–121.
5. Suzuki M, Takatsuki F, Maeda YY, Hamuro J, Chihara G. Lentinan. Rationale for development and therapeutic potential. *Clin Immunother* 1994; **2**: 121–133.
6. Morinaga H, Tazawa K, Tagoh H, Muraguchi A, Fujimaki M. An *in vivo* study of hepatic and splenic interleukin-1 beta mRNA expression following oral PSK or LEM administration. *Jpn J Cancer Res* 1994; **85**: 1298–1303.
7. Maeda YY, Yonekawa H, Chihara G. Application of lentinan as cytokin inducer and host defense potentiator in immunotherapy of infectious diseases. In: Masihi N, ed. *Immunotherapy of Infections*. New York, Basel, Hong Kong: M. Dekker, 1994; 261–279.
8. Szabo T, Kadish JL, Czop JK. Biochemical properties of the ligand-binding 20-kDa subunit of the β-glucan receptors on human mononuclear phagocytes. *J Biol Chem* 1995; **270**: 2145–2151.
9. Thornton BP, Vetvicka V, Pitman M, Goldman RC, Ross GD. Analysis of the sugar specificity and molecular location of the β-glucan-binding lectin site of complement receptor type 3 (CD11b/CD18). *J Immunol* 1996; **156**: 1235–1246.
10. Muller A, Rice PJ, Ensley HE, Coogan PS, Kalbfleisch JH, Kelly JL, *et al.* Receptor binding and internalization of water-soluble (1 → 3)-β-D-glucan biologic response modifier in two monocyte/macrophage cell lines. *J Immunol* 1996; **156**: 3418–3425.
11. Duan XM, Ackerly E, Vivier E, Anderson P. Evidence for involvement of β-glucan-binding cell surface lectins in human natural killer cell function. *Cell Immunol* 1994; **157**: 393–398.
12. Muller CD, Bocchini V, Giaimis J, Guerrieri P, Lombard Y, Poindron P. Functional beta-glucan receptor expression by a microglial cell line. *Res Immunol* 1994; **145**: 267–275.
13. Tapper H, Sundler R. Glucan receptor and zymosan-induced lysosomal enzyme secretion in macrophages. *Biochem J* 1995; **306**: 829–835.
14. Okazaki M, Adachi Y, Ohno N, Yadomae T. Structure-activity relationship of (1 → 3)-beta-D-glucans in the induction of cytokine production from macrophages, *in vitro*. *Biol Pharm Bull* 1995; **18**: 1320–1327.
15. Okazaki M, Chiba N, Adachi Y, Ohno N. Signal transduction pathway on β-glucans-triggered hydrogen peroxide production by murine peritoneal macrophages *in vitro*. *Biol Pharm Bull* 1996; **19**: 18–23.
16. Franz G, Alban S. Structure-activity relationship of antithrombotic polysaccharide derivatives. *Int J Macromol* 1995; **17**: 311–314.
17. Alban S, Jeske W, Welzel D, Franz G, Fareed J. Anticoagulant and antithrombotic actions of a semisynthetic beta-1,3-glucan sulfate. *Thromb Res* 1995; **78**: 201–210.
18. Konopski Z, Fandrem J, Seljelid R, Eskeland T. Interferon-gamma inhibits endocytosis of soluble aminated beta-1,3-D-glucan and neutral red in mouse peritoneal macrophages. *J Interferon Cytokine Res* 1995; **15**: 579–603.
19. Yamamoto N, Nagashima H, Yoshida O, Kaneko Y, Matsuzaki K, Uryu T. Effect of the sulfated polysaccharides on HIV: a novel strategy of chemical modification for HIV antivirals. *Arch AIDS* 1988; **1**: 45–56.
20. Jagodzinski PP, Wiaderkiewicz R, Kurzawski G, Kloczewiak M, Nakashima H, Hyjek E, *et al.* Mechanism of the inhibitory effect of curdlan sulphate on HIV-1 infection *in vitro*. *Virology* 1994; **202**: 735–745.
21. Gordon M, Guralnik M, Kaneko Y, Mimura T, Baker M, Lang W. A phase I study of curdlan sulphate—an HIV inhibitor. Tolerance, pharmacocinetics and effects on coagulation and on CD4 lymphocytes. *J Med* 1994; **25**: 163–180.
22. Gordon M, Guralnik M, Kaneko Y, Mimura T, Goodgame J, Lang W. Further clinical studies of curdlan sulphate (CRDS) an anti HIV agent. *J Med* 1995; **26**: 97–131.
23. Alban S, Franz G. Gas liquid chromatography—mass spectrometry analysis of anticoagulant active curdlan sulfate. *Semin Thromb Hemost* 1994; **20**: 152–158.
24. Kanke M, Katayama H, Nakamura M. Application of curdlan to controlled drug delivery. *Biol Pharm Bull* 1995; **18**: 1104–1108, 1154–1158.
25. Koj A, Gauldie J, Sweeney GD, Regoeczi E, Sauder DN. The acute phase response of cultured rat hepatocytes. System characterization and the effect of human cytokines. *Biochem J* 1984; **224**: 505–515.
26. Rose-John S, Dietrich A, Marks F. Molecular cloning of mouse protein kinase C (PKC) cDNA from Swiss 3T3 fibroblasts. *Gene* 1988; **74**: 465–471.
27. Feinberg AP, Vogelstein B. A technique for radiolabeling DNA restriction endonuclease fragments to high specific activities. *Analyt Biochem* 1983; **132**: 6–13.
28. Suzuki YJ, Mizuno M, Packer L. Signal transduction for nuclear factor-κB activation. Proposed location of antioxidant-inhibitable step. *J Immunol* 1994; **153**: 5008–5015.
29. Duyao MP, Buckler AJ, Sonenshein GE. Interaction of an NK-κB-like factor with a site upstream of the c-myc promoter. *Proc Natl Acad Sci* 1990; **87**: 4727–4731.
30. Maire P, Wuarin J, Schibler U. The role of cis-acting promoter elements in tissue-specific albumin gene expression. *Science* 1989; **244**: 343–345.
31. Koj A, Korzus E, Baumann H, Nakamura T, Travis J. Regulation of synthesis of some proteinase inhibitors in human hepatoma cells by cytokines, hepatocyte growth factor and retinoic acid. *Biol Chem Hoppe-Seyler* 1993; **374**: 193–201.
32. Malawista SE, Van Damme J, Sipe JD, Duff GW, Weiss MC. Independent effects of interleukin-6 (IL-6) and interleukin-1 (IL-1) on accumulation of specific mRNA for fibrinogen and for serum amyloid A in human hepatoma (HepG2) cells. *Ann NY Acad Sci* 1989; **557**: 518–520.
33. Rokita H, Bartle L, Sipe J. Differential effect of interleukin-1 on interleukin-6-stimulated alpha-1-antichymotrypsin expression in human hepatoma cell lines. *Folia Histochem Cytobiol* 1992; **30**: 161–164.
34. Sehgal PB, Wang L, Rayanade R, Pan H, Margulies L. Interleukin-6-type cytokines. *Ann NY Acad Sci* 1995; **762**: 1–14.
35. Koj A. The role of interleukin-6 as the hepatocyte stimulating factor in the network of inflammatory cytokines. *Ann NY Acad Sci* 1989; **557**: 1–8.
36. Kopf M, Ramsay A, Brombacher F, Baumann H, Freer G, Galanos Ch, Gutierrez-Ramos J-C, Kohler G. Pleiotropic defects of IL-6-deficient mice including early hematopoiesis, T and B cell function, and acute phase responses. *Ann NY Acad Sci* 1995; **762**: 308–318.
37. Liao W S-L, Lu S-Y, Huang J, Li L, Bing Z. Transcription factors as targets of cytokine signals. *Ann NY Acad Sci* 1995; **762**: 238–251.
38. Chen H-M, Liao WSL. Differential acute-phase response of rat kininogen genes involves type I and type II interleukin-6 response elements. *J Biol Chem* 1993; **268**: 25311–25319.
39. Mizuguchi J, Hu Ch-H, Cao Z, Loeb KR, Chung DW, Davie EW. Characterization of the 5′-flanking region of the gene for the γ chain of human fibrinogen. *J Biol Chem* 1995; **270**: 28350–28356.
40. Shimizu H, Yamamoto K. NF-kappa B and C/EBP transcription factor families synergistically function in mouse serum amyloid A gene expression induced by inflammatory cytokines. *Gene* 1994; **149**: 305–310.
41. Maeda Y, Chihara Y, Ishimura K. Unique increase of serum proteins and action of antitumor polysaccharides. *Nature* 1974; **252**: 250–252.
42. Maeda Y, Takahama S, Kohara Y, Yonekawa H. Two genes controlling acute phase responses by the antitumor polysaccharide, lentinan. *Immunogenetics* 1996; **43**: 215–219.
43. Fridovich I. Superoxide radical and superoxide dismutases. *Annu Rev Biochem* 1995; **64**: 97–112.
44. Wan XS, Devalaraja MN, StClair DK. Molecular structure and organization of the human manganese superoxide dismutase gene. *DNA Cell Biol* 1994; **13**: 1127–1136.
45. Borello S, DeLeo ME, Landriscina M, Palazzotti B, Galeotti T. Diethyldithiocarbamate treatment up regulates manganese superoxide dismutase gene expression in rat liver. *Biochem Biophys Res Commun* 1996; **220**: 546–552.

ACKNOWLEDGEMENTS. We are grateful to Anjinomoto Co., Inc. for a generous gift of curdlan sulphate, and to Dr H. Baumann for IL-6. We thank Dr A. Koj (Institute of Molecular Biology, Jagiellonian University) for valuable criticism and suggestions and Dr J. Bereta for help with MnSOD mRNA estimation.

**Received 24 October 1996;**
**accepted in revised form 27 November 1996**
